# Enhanced Amitriptyline Degradation by Electrochemical Activation of Peroxydisulfate: Mechanisms of Interfacial Catalysis and Mass Transfer

**DOI:** 10.3390/molecules30183835

**Published:** 2025-09-22

**Authors:** Teer Wen, Fangying Hu, Yao Yue, Chuqiao Li, Yunfei He, Jiafeng Ding

**Affiliations:** 1 School of Engineering, Hangzhou Normal University, Hangzhou 310018, China; 2023111010099@stu.hznu.edu.cn (T.W.); 2024111032004@stu.hznu.edu.cn (F.H.); 2023111010067@stu.hznu.edu.cn (Y.Y.); 2022111010069@stu.hznu.edu.cn (C.L.); 2 Zhejiang Provincial Key Laboratory of Wetland Intelligent Monitoring and Ecological Restoration, Hangzhou 311121, China

**Keywords:** amitriptyline, persulfate, C@ZnO electrode, ROS, mass transfer

## Abstract

Amitriptyline (AMT), a widely prescribed antidepressant, and its metabolites have emerged as significant environmental contaminants, posing substantial risks to aquatic organisms and human health. Systematic and in-depth investigations into advanced anode materials, coupled with a profound elucidation of their electrochemical mechanisms, are imperative for the development of efficacious technologies for AMT removal. In this study, a series of amorphous carbon-encapsulated zinc oxide (C@ZnO) modified anodes were systematically synthesized and incorporated into a persulfate-based electrochemical system (CZ-PS) to comprehensively elucidate the catalytic mechanisms and mass transfer efficiencies governing the degradation of AMT via electroperoxidation. Notably, the CZ-PS system achieved a 97.5% degradation for 5.0 mg/L AMT within 120 min under optimized conditions (200 C@ZnO electrode, pH 7.0, current density 20 mA/cm^2^, PS concentration 0.5 mM), significantly outperforming the single PS system (37.8%) or the pure electrocatalytic system. Quenching experiments and EPR analysis confirmed hydroxyl radicals (•OH) and sulfate radicals (SO_4_•^−^) as the dominant reactive species. Both acidic and neutral pH conditions were demonstrated to favorably enhance the electrocatalytic degradation efficiency by improving adsorption performance and inhibiting •OH decomposition. The system retained >90% degradation efficiency after 5 electrode cycles. Three degradation pathways and 13 intermediates were identified via UPLC–MS/MS analysis, including side-chain demethylation and oxidative ring-opening of the seven-membered ring to form aldehyde/carboxylic acid compounds, ultimately mineralizing into CO_2_ and H_2_O. It demonstrates strong engineering potential and provides a green, high-efficiency strategy for antibiotic wastewater treatment.

## 1. Introduction

Antibiotics in pharmaceuticals and personal care products (PPCPs) have garnered significant attention due to their persistence, bioaccumulative potential, and ecotoxicity [1,2,3]. The antibiotic wastewater primarily originates from hospitals, pharmaceutical manufacturers, and livestock farms [4,5,6]. The release of large quantities of antibiotics into the environment inevitably exerts negative impacts on non-target organisms within ecosystems. Thus, the effective elimination of antibiotics from aquatic environments has emerged as a critical research focus. Amitriptyline (AMT), a tricyclic antidepressant, is clinically used for treating enuresis, migraines, and diabetic peripheral neuropathic pain [7,8]. AMT is frequently detected in environmental matrices, with concentrations exceeding 500 ng L^−1^ in certain regions [9]. Consequently, developing effective technologies for treating AMT and other antibiotics and PPCPs in wastewater is of significant importance.

To enhance the removal efficiency of AMT from aqueous environments, several emerging strategies have been developed and optimized in recent research endeavors, including biodegradation, metal oxide oxidation, advanced oxidation processes (AOPs), electrocatalytic oxidation, photocatalysis, and metal/bimetallic reduction [10,11,12,13,14,15,16]. For instance, Tee et al. [11] successfully synthesized boron-doped three-dimensional (3D) graphene composite materials, achieving outstanding adsorption performance on AMT removal through optimized surface functionalization and porous architecture design. Concurrently, Finčur et al. [16] reported a novel heterogeneous photocatalytic system utilizing TiO_2_/WO_3_ composite coatings, which exhibited enhanced charge separation efficiency and visible light responsiveness to improved oxidative degradation kinetics of AMT pollutants. Although these methods demonstrate strong degradation capabilities, the toxicity of primary intermediates and byproducts from AMT degradation remains largely unknown. Carbon materials (e.g., carbon nanotubes, graphene, and amorphous carbon) with high electrical conductivity and light absorption intensity have attracted extensive attention in recent years.

Zinc oxide nanoparticles (ZnO), as a promising n-type semiconductor material, exhibit a zeta potential of 16 mV in colloidal particles and approximately 27 clusters in ZnO nanoparticles [17,18,19]. This charge-driven aggregation enables feasible coagulation–sedimentation for organic contaminants. Furthermore, anchoring ZnO onto carbon matrices (e.g., reduced graphene oxide) to form heterostructures effectively mitigates nanoparticle agglomeration by reducing electrostatic repulsion while enhancing interfacial charge transfer efficiency [20,21]. In electrocatalytic systems, carbon materials (e.g., biochar) act as highly conductive scaffolds that promote expedited electron shuttling through their interconnected three-dimensional networks, thereby endowing the system with enhanced catalytic efficacy and stability under electrochemical conditions [22]. Recently, oxidation processes based on persulfates (e.g., peroxymonosulfate HSO_5_^−^/PMS, peroxydisulfate S_2_O_8_^2−^/PDS) have been widely applied in water pollution treatment. Serving as stable, low-cost, and safe oxidant carriers, they form Fenton-like systems with transition metals for pollutant degradation [23,24,25]. However, practical limitations in natural water matrices often reduce their efficiency. Thus, integrating complementary systems to enhance performance while reducing energy consumption has become research priority [26,27].

In this study, a novel series of amorphous C@ZnO composites were synthesized via a simple impregnation–calcination method and integrated into a customized anode configuration within the CZ-PS electrocatalytic system for AMT degradation. The morphology, composition, optical properties, and stability of C@ZnO were characterized. Batch experiments were conducted to elucidate the effects of PS dosage, pH, and initial AMT concentration on the degradation performance. Mechanistic investigations were further deepened through selective radical scavenging and EPR spectroscopy, confirmed ROS-mediated mineralization. Notably, intermediate studies elucidated the degradation pathways and mechanisms of AMT in the electrocatalytically activated PS system. This work pioneers an innovative paradigm for the rational design of high-efficiency electrocatalysts, holding significant promise for advanced antibiotic remediation in practical wastewater treatment scenarios.

## 2. Results and Discussion

### 2.1. Structural Characteristics of C@ZnO Materials

**Figure 1:**
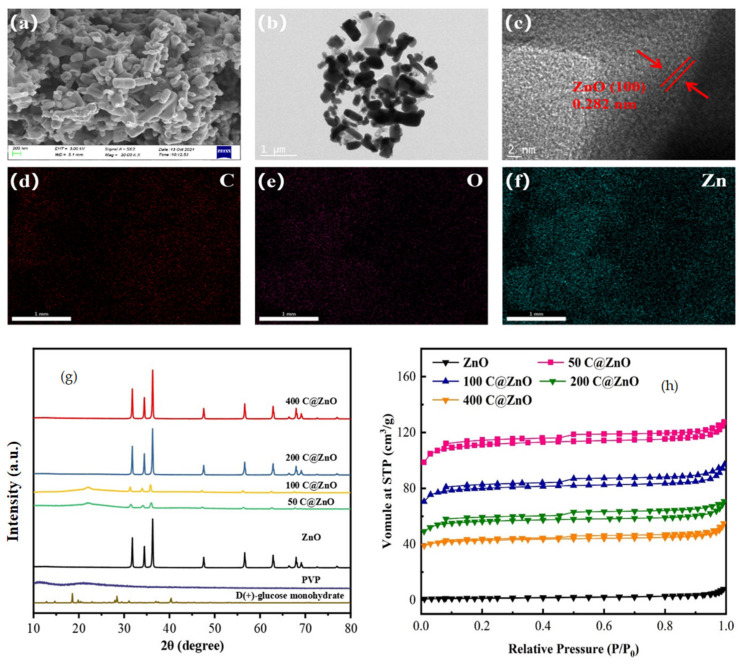
Characterization of C@ZnO (**a**) SEM, (**b**,**c**) TEM, (**e**) HRTEM, and (**d**–**f**) EDS images, (**g**) XRD pattern of C@ZnO, and (**h**) the N_2_ adsorption–desorption isotherm of ZnO and C@ZnO.

The surface morphology and elemental composition of the material samples were characterized by SEM, EDS, and TEM, as shown in Figure 1. The C@ZnO samples exhibited a grayish-black color and a columnar structure with a rough surface, closely resembling the morphology of pristine ZnO nanoparticles (Figure 1a). This observation confirmed the formation of a composite structure in which amorphous carbon encapsulates ZnO. TEM images revealed tightly bound ZnO particles within the carbon matrix. High-resolution TEM analysis of C@ZnO (Figure 1b,c) showed an interplanar spacing of 0.282 nm, corresponding to the (100) plane of ZnO. According to previous studies, the (0001) basal plane of ZnO possesses high surface energy in a polarized state, potentially influencing its interaction with the carbon coating [28]. EDS analysis and elemental mapping (Figure 1d–f) of 200 C@ZnO demonstrated homogeneous distribution of C, O, and Zn elements across the surface, indicating successful integration of the composite components.

X-ray diffraction (XRD) patterns (Figure 1g) verified the crystalline phase of C@ZnO composites. Characteristic peaks corresponding to the (100), (002), (101), (102), (110), (103), (200), (112), and (201) planes of ZnO were observed at 31.93°, 34.68°, 36.44°, 47.74°, 56.75°, 63.0°, 66.5°, 68.06°, and 69.3°, respectively, consistent with the standard ZnO reference (JCPDS No. 36-1451). Increasing ZnO content enhanced diffraction peak intensity. The absence of PVP/glucose diffraction peaks indicated phase transformation during synthesis, with residual peaks potentially serving as background signals [21]. BET/BJH analysis of N_2_ adsorption–desorption isotherms (Figure 1h) confirmed Type IV isotherms (IUPAC classification) for all samples, signifying mesoporous capillary condensation systems.

Hysteresis loops exhibited H4-type behavior, indicating low pore structure regularity without distinct saturation adsorption. As summarized in Table 1, specific surface area decreased sequentially: 443.44 m^2^ g^−1^ (50 C@ZnO) > 319.45 m^2^ g^−1^ (100 C@ZnO) > 224.30 m^2^ g^−1^ (200 C@ZnO) > 169.20 m^2^ g^−1^ (400 C@ZnO) > 4.04 m^2^ g^−1^ (pristine ZnO); the amorphous carbon coating dramatically increased specific surface area (e.g., 4.04 → 224.30 m^2^ g^−1^ for pristine ZnO vs. 200 C@ZnO). This enhanced porosity and surface area directly correlated with superior adsorption capacity compared to pristine ZnO.

### 2.2. Surface Structure of C@ZnO Materials

**Figure 2:**
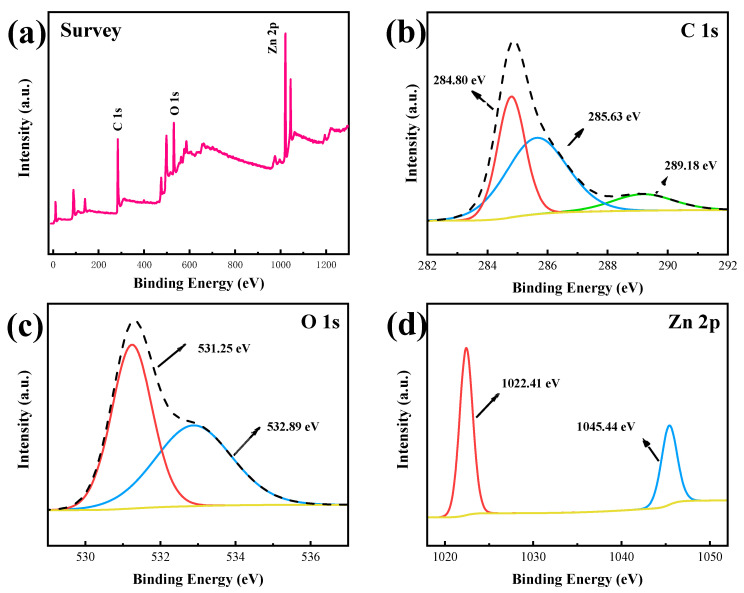
XPS spectra of 200 C@ZnO: (**a**) Survey, (**b**) C1 s, (**c**) O1 s, and (**d**) Zn 2p.

The elemental composition of the material sample 200 C@ZnO was characterized by XPS, and the results are shown in Figure 2. As shown in Figure 2a, the 200 C@ZnO sample revealed the presence of Zn, O, and C as major elements. The high-resolution C 1s spectrum (Figure 2b) showed peaks at 284.80 eV (C-C), 285.63 eV (C-O), and 289.18 eV (O-C=O), indicating the formation of carbon bonds [29]. The O 1s spectrum (Figure 2c) exhibited peaks at 531.25 eV (Zn-O) and 532.89 eV (C-O), attributed to lattice oxygen in ZnO and surface hydroxyl/chemisorbed oxygen, respectively [30]. The high-resolution XPS spectrum of Zn 2p for the sample (Figure 2d) exhibited two characteristic peaks at 1022.4 eV (Zn 2p_3/2_) and 1045.4 eV (Zn 2p_1/2_), consistent with the binding energies of Zn-O bonds in ZnO. The ~23 eV separation between these peaks, arising from spin-orbit coupling, matched the characteristic Zn 2p doublet spacing reported in previous studies, confirming the presence of ZnO in its crystalline phase [31].

**Figure 3:**
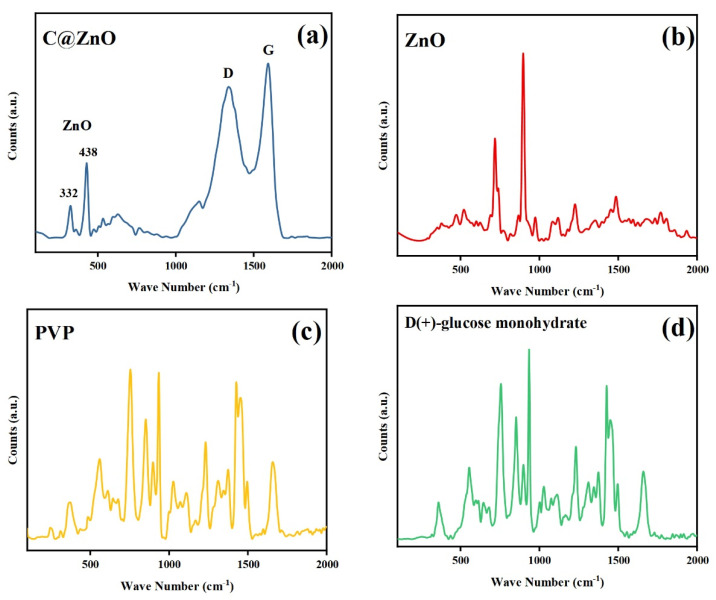
Raman spectra of 200 C@ZnO (**a**), ZnO (**b**) and the synthetic raw: polyvinylpyrrolidone (**c**), D(+)-Glucose monohydrate (**d**).

Raman spectroscopy was employed to characterize the crystal structure of the sample under ambient conditions, with results shown in Figure 3. The Raman spectrum of 200 C@ZnO (Figure 3a) exhibited two characteristic peaks at 332 cm^−1^ and 438 cm^−1^, assignable to the Zn-O stretching modes, consistent with XRD data. Additionally, peaks at 1340 cm^−1^ (D band) and 1590 cm^−1^ (G band) were observed, indicative of carbonaceous components (e.g., graphene-like structures or disordered carbon). According to previous reports, ZnO exhibits Raman-active optical phonons of A_1_ + E_1_ + 2E_2_ symmetry, with A_1_ and E_1_ modes capable of splitting into transverse optical (TO) and longitudinal optical (LO) branches, respectively [32]. The presence of Zn-O peaks and carbon-related bands collectively confirms the composite nature of the material. As depicted in Figure 3b, the Raman spectrum of pristine ZnO exhibits characteristic peaks at 332 cm^−1^ and 438 cm^−1^, corresponding to the Zn-O stretching modes of the B4 phase, which confirm its hexagonal wurtzite structure [28]. In contrast, the C@ZnO spectrum lacks the derivative peaks associated with PVP and glucose (Figure 3c,d), indicating that these precursor materials underwent complete transformation during synthesis, preventing their spectral signatures from being detected. The additional bands observed in C@ZnO likely arise from the amorphous carbon layer formed on the surface.

### 2.3. Degradation Performance and Optimization

**Figure 4:**
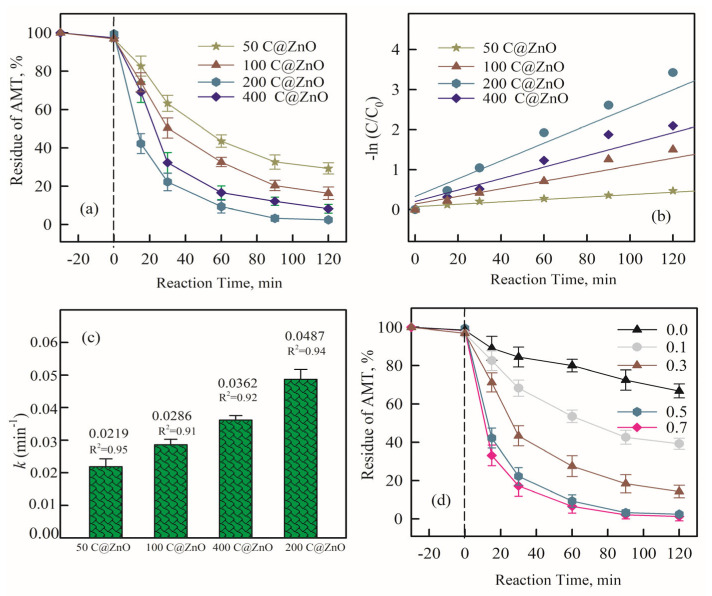
The impact of initial ZnO concentration (mg of ZnO) in C@ZnO material on AMT removal (**a**), the pseudo-first-order rate constant of AMT removal by different C@ZnO material in AMT removal (**b**,**c**), and initial PS concentration (**d**, mM) on AMT removal in 200 C@ZnO material system after 120 min. CNa_2_SO_4_ = 5 mM, current density = 20 mA/cm^2^, pH = 7.0, and CAMT = 5 mg/L. Data points for AMT residue were means ± standard deviations (n = 3).

To investigate the degradation efficiency of AMT in different electrocatalytic oxidation systems, 5 mg/L AMT was degraded for 120 min using persulfate (PS) alone, the electrochemical (EC) system alone, and the C@ZnO anode (CZ-PS) system. As shown in Figure 4a, the electrode materials used in the CZ-PS system were 50 C@ZnO, 100 C@ZnO, 200 C@ZnO, and 400 C@ZnO. As the ZnO concentration increased from 50 mg to 200 mg, the AMT removal rate in the CZ-PS system significantly improved (78.9%, 83.4%, and 97.4%, respectively), demonstrating the crucial role of ZnO in the system. However, when the ZnO concentration reached 400 mg, the degradation efficiency slightly decreased, likely due to the blocking of the semiconductor’s active sites, which hindered the generation of free radicals [33,34].

The first-order kinetic reaction model of different systems to the degradation of AMT is shown in Figure 4b. The first-order kinetic reaction model was also improved in the same order [35]: ln (C/C_0_) = −kt. Additionally, the half-lives (t_1/2_) were calculated by: t_1/2_ = ln2/k. Obviously, the catalytic efficiency was enhanced with the addition of ZnO concentration, and the corresponding k values increased from 0.0219 min^−1^ to 0.0489 min^−1^. This significant improvement is owing to a better decrease in SO_4_^•−^, ^•^OH, and ^1^O_2_ generation.

As shown in Figure 4b, compared to the EC system using only the C@ZnO anode, the CZ-PS system exhibited a significantly enhanced AMT removal rate. Within 120 min, the system containing only PS degraded 37.8% of AMT. The degradation performance of the electrocatalytic system with the 200 C@ZnO anode increased markedly. As the PS concentration increased from 0.1 mM to 0.7 mM, the removal rate rose from 60.2% to 100%. This can be attributed to the direct oxidation of AMT on the C@ZnO anode and the oxidation by reactive species (ROS) generated via hydrolysis [34]. Since the generated reactive species adsorb onto the anode, the removal rate in the CZ-PS system increased to 97.5% within 120 min after adding PS. Notably, elevated PMS concentrations may result in suppressed pollutant removal efficiency, attributed to the scavenging of generated ROS [36,37]. This phenomenon indicates a significant synergistic effect when PS is combined with electrochemical oxidation.

### 2.4. Influence of Reaction Conditions

**Figure 5:**
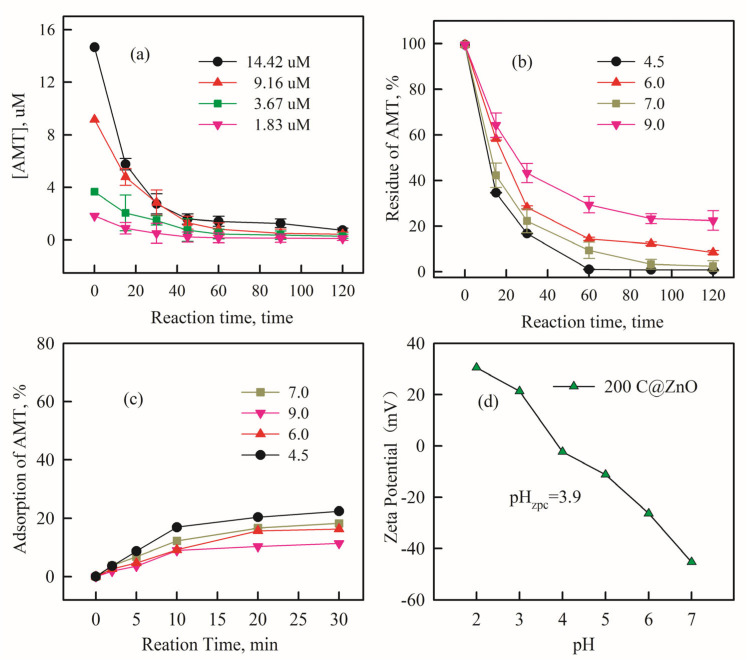
The impact of initial AMT concentration (**a**) and initial pH (**b**) on AMT removal in system after 120 min; the sorption properties of AMT change with the different pH values after 30 min (**c**); and Zeta potential of 200 C@ZnO material under various pH conditions (**d**). CNa_2_SO_4_ = 5 mM, current density = 20 mA/cm^2^, and C_PS_ = 0.5 mM.

Figure 5a illustrates the effect of different initial AMT concentrations on the degradation process (CZ-PS system). The AMT removal rate slightly increased as the initial AMT concentration decreased. After 120 min of degradation, the removal rate was 97.4% at an initial concentration of 14.42 μM, while it reached 98.1% and 99.5% at concentrations of 9.16 μM and 3.67 μM, respectively. The removal rate improved as the initial AMT concentration decreased from 14.42 μM to 3.67 μM, and at an initial concentration of 1.83 μM, AMT was almost completely removed (near 100%). Clearly, a higher pollutant concentration corresponds to a lower removal rate. This phenomenon may be attributed to the generation of more intermediates at higher concentrations [14]. These intermediates diffuse and adsorb onto the anode surface, competing with the pollutant for active sites and Reactive oxygen species (ROS) [38,39], thereby reducing the pollutant removal efficiency.

The initial pH of the solution significantly impacts the degradation process because H^+^ participates in numerous free radical chain reactions [40]. Experiments investigated the effect of initial pH (ranging from 4.5 to 9) on the electrocatalytic degradation of AMT using the 200 C@ZnO anode. As shown in Figure 5b, the AMT removal rate was higher under acidic conditions compared to alkaline conditions. Furthermore, the removal rate reached its maximum at pH 4.5, indicating that acidic conditions favor antibiotic degradation. As can be seen from Figure 5c, it demonstrates that during the adsorption process, pH values ranging from acidic to neutral significantly facilitate the adsorption of amitriptyline onto the catalyst surface, achieving adsorption efficiencies of approximately 10–20%. When the solution is alkaline, electron transfer at the C@ZnO anode surface is impeded, reducing electron generation [41]. Conversely, under acidic conditions, the presence of H^+^ inhibits the decomposition of ·OH into oxygen (OH + H_2_O → O_2_ + 3H^+^ + 3e^−^) [42], thereby enhancing the utilization efficiency of ·OH and increasing pollutant removal efficiency. Based on the Zeta potential measurements in Figure 5d, the zero charge point (ZPC) of the C@ZnO anode was determined to be 3.9. At elevated pH values, the electrode surface develops a pronounced negative potential, which enhances the electrostatic repulsion toward persulfate and sulfate ions. Consequently, this hampers the adsorption of pollutants onto the electrode, resulting in diminished removal efficiency. At lower pH values, the N lone pair electrons of the amine group undergo protonation, forming a positively charged ammonium moiety. This protonation significantly hampers electron donation from the amino group, thereby impeding electron transfer processes. Conversely, during deprotonation, the recovery of the amine group’s electron-donating capacity facilitates redox reactions, enabling more efficient degradation of pollutants [43]. Additionally, the energy consumption for AMT degradation was relatively lower at pH 7. Hence, pH 7 was selected for subsequent degradation application studies.

**Figure 6:**
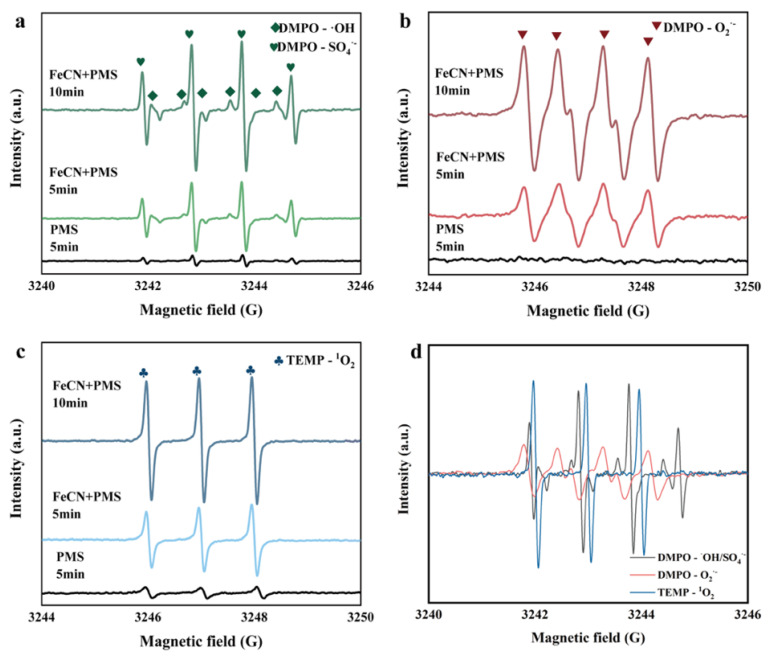
The EPR spectrum of SO_4_^•−^ and ^•^OH (**a**), O_2_^•−^ (**b**), ^1^O_2_ (**c**), The intensity increase of ROS from 5 to 10 min (**d**).

Electron paramagnetic resonance (EPR) spectroscopy was employed to characterize the reactive oxygen species (ROS) generated by the CZ-PS system. As shown in Figure 6a, distinct signals corresponding to DMPO-SO_4_^•−^ and DMPO-^•^OH radicals were detected, indicating the formation of sulfate (SO_4_^•−^) and hydroxyl radicals (^•^OH), respectively. Furthermore, Figure 6b revealed the characteristic signals of O_2_^•−^, while a 1:1:1 triplet signal attributed to TEMP-^1^O_2_ was observed in Figure 6c. Notably, the intensities of all ROS signals exhibited a time-dependent increase between 5 and 10 min (Figure 6d), suggesting continuous generation of SO_4_^•−^, ^•^OH, O_2_^•−^, and ^1^O_2_ within the system. These findings collectively demonstrate the efficacy of this system as a sustainable ROS generator, paving the way for its application in PPCPs removal experiments.

**Figure 7:**
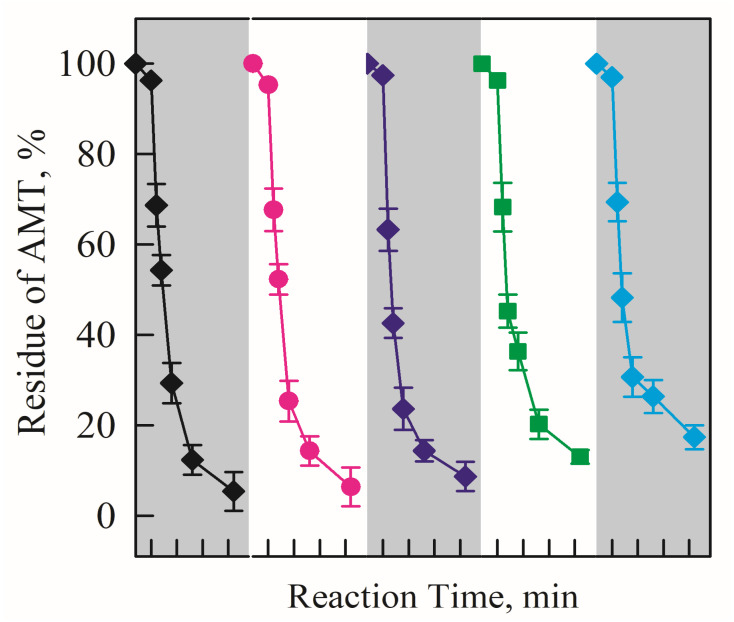
AMT was degraded over five consecutive cycles using the electrocatalytic system with the 200 C@ZnO anode.

To investigate the stability and reusability of the electrode, the same piece of C@ZnO anode was used to degrade AMT for 5 consecutive cycles under identical operating conditions. As shown in Figure 7, the AMT removal rate using the C@ZnO anode remained above 90% throughout all cycles. The degradation efficiency showed almost no reduction after five reuses, demonstrating the excellent electrocatalytic activity and stability of the C@ZnO anode.

### 2.5. Degradation Pathway and Reaction Mechanism

**Figure 8:**
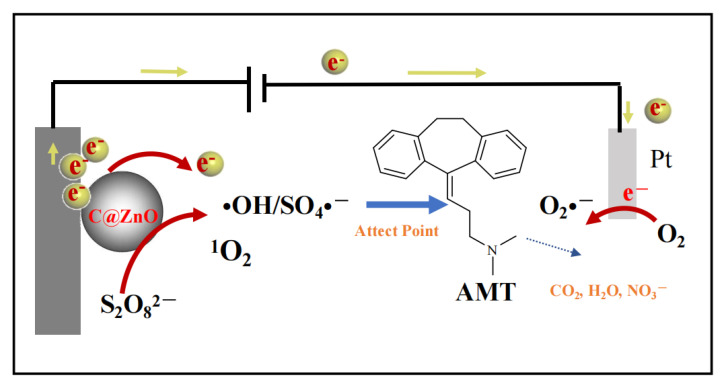
Proposed schematic illustration of the mechanism for degradation of AMT in the CZ-PS system.

**Figure 9:**
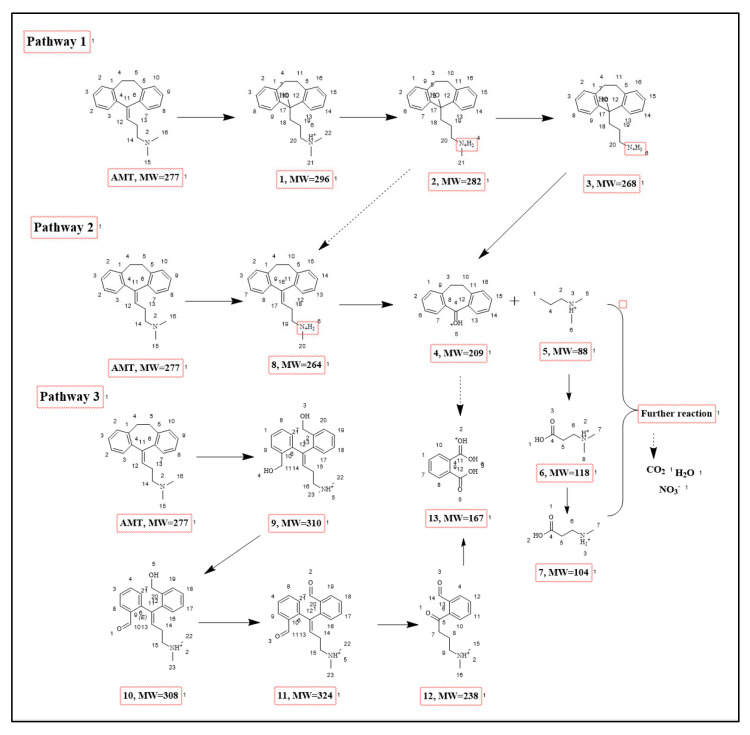
Proposed Degradation Pathway of AMT in the CZ-PS System.

In the CZ-PS system, the generation pathways of O_2_^•−^, ^•^OH, SO_4_^•−,^ and ^1^O_2_ are depicted in Figure 8 and Figure 9 as follows. In persulfate-mediated reactions, the disruption of -O-O- bonds in persulfate can lead to the formation of free radicals such as ^•^OH and sulfate SO_4_^•−^ [40]. The continuous redox cycling between two valence states of Zn ions on the surface enables the potential generation of ^1^O_2_ via 2 distinct mechanistic pathways: either through direct oxidation and subsequent recombination of O_2_^•−^, or through the oxidative transformation of SO_4_^•−^ [41].

To further reveal the primary transformation products formed during AMT degradation in the C@ZnO anode/PS system, UPLC–MS/MS was employed for their identification. As can be seen from Figure 9, the degradation of AMT primarily proceeds through three distinct pathways, involving a total of 13 intermediates. Sequential demethylation and hydroxylation reactions initially modify the AMT molecule, yielding intermediates Product 1, Product 2, and Product 3 [42]. Subsequently, the dimethylamine moiety in Product 3 undergoes further demethylation and oxidative transformations, generating Products 4 and 5 [44]. Finally, Product 13 is formed via the cleavage of side chains from both Product 4 and Product 5, completing the degradation cascade. Then, Product 6 and Product 7 are formed via continuous ring-opening and hydroxylation reactions attacked by ROS; the seven-membered ring of the AMT molecule is opened through oxidation and hydroxylation, generating intermediate Product 9 [45]. The alcoholic hydroxyl group in Product 9 is continuously oxidized to an aldehyde, forming Product 10 and Product 11. Product 11 can also react with ·OH and sulfate radicals SO_4_^•−^ to yield Product 12. Subsequently, Product 11 and Product 12 undergo aldehyde oxidation and alcoholic hydroxyl oxidation reactions, respectively, forming Product 13, where the aldehyde is further converted into a carboxyl group; Product 6 and Product 7 are also obtained through the cleavage of the side chain from Product 3. These aromatic intermediates are then further oxidized and mineralized into small molecules (such as NO_3_^−^, H_2_O, and CO_2_) via continuous ring-opening and hydroxylation reactions attacked by ROS.

## 3. Materials and Methods

### 3.1. Chemicals and Reagents

Amitriptyline (AMT, 98%), zinc oxide (ZnO, 99%), and polyvinylpyrrolidone ((C_6_H_9_NO)_n_, K23-27) were purchased from Aladdin (Shanghai, China) and Macklin (Shanghai, China), respectively. D(+)-Glucose monohydrate (C_6_H_12_O_6_·H_2_O, 98%), ethanol (C_2_H_6_O, ≥99.7%), potassium chloride (KCl, ≥99.5%), and nitric acid (HNO_3_, 65.0–68.0%) were all obtained from Sinopharm Chemical Reagent Co., Ltd. (Shanghai, China). All chemicals were of reagent grade or higher purity and were used without further purification. All aqueous solutions used in the experiments were prepared using deionized water.

### 3.2. Synthesis of C@ZnO Catalysts

Carbon-encapsulated zinc oxide nanocomposites (C@ZnO) were synthesized using a hydrothermal–calcination method [21]. Specifically, 0.5, 1.0, 2.0, and 4.0 g of ZnO were mixed with equal amounts of PVP in 300 mL of ultrapure water. Then, 19.8 g of glucose was added to the above suspension. After 24 h of stirring, the suspension was hydrothermally treated at 180 °C for 4 h, washed with ethanol, and dried at 60 °C. The resulting powder was calcined under N_2_ at 600 °C (10 °C/min ramp, 4 h hold), yielding dark grey solid powder. This powder was ground and sieved through a 60-mesh nylon sieve to ensure uniform particle size. Through this process, amorphous carbon-encapsulated ZnO composites were obtained, with ZnO masses of 50 mg, 100 mg, 200 mg, and 400 mg incorporated during synthesis. These were designated as 50 C@ZnO, 100 C@ZnO, 200 C@ZnO, and 400 C@ZnO, respectively.

### 3.3. Electrode Preparation and Screening

The C@ZnO-modified anode was fabricated via spin-coating of active material powder onto a dopant layer prepared on fluorine-doped tin oxide (FTO) glass. Prior to modification, the FTO substrates (50 mm × 40 mm × 2 mm) were successively rinsed with acetone and deionized water to remove surface impurities and oxide films. Following a 15-min ultrasonic cleaning step, 30 mg of C@ZnO powder was dispersed in a 1.5 mL mixture of ethanol and naphthol (1:1 *v*/*v*), subjected to ultrasonic treatment for 30 min to ensure homogeneous suspension. The resultant colloidal dispersion was uniformly coated onto the FTO substrate under infrared irradiation and subsequently dried overnight in a vacuum oven at 60 °C, yielding the C@ZnO-modified electrode.

### 3.4. Screening Experiments for Modified Electrodes

Electrocatalytic oxidation experiments were performed under the following conditions: current density of 20 mA/cm^2^, electrode spacing of 20 mm, initial AMT concentration of 5 mg/L, and 5 mM sodium sulfate added as the supporting electrolyte. Through these screening experiments, the modified C@ZnO electrode exhibiting the best electrocatalytic activity was identified. Before powering, the suspension was magnetically stirred for 30 min in dark in order to obtain a homogeneous mixture. This selected electrode was then used in subsequent experiments to investigate the factors influencing AMT removal from water. Using the screened modified electrode as the anode and Pt as the cathodes, with 5 mM Na_2_SO_4_ as the supporting electrolyte and an electrode spacing of 20 mm, the effects of different initial AMT concentrations, different current densities, and different initial pH values on the electrocatalytic oxidation removal efficiency of AMT from water were examined. Samples were taken every 30 min during the 120-min reaction period. To evaluate stability and reusability in experiments (e.g., sensor performance, catalyst efficiency, material durability), the Relative Standard Deviation (RSD) is essential for quantifying precision and consistency [46]. Here’s why and how it’s used:

RSD (%) = (Standard Deviation (SD)/Mean) × 100%(1)

In these conditions, the treated samples were incubated at 25 ± 1 °C. At designated time intervals, triplicate samples (one centrifuge tube for each sample) were withdrawn for the total AMT residue analysis. All of the experiments were treated in triplicate. Data points for AMT residue were means ± standard deviations (n = 3).

### 3.5. Analytical Methods

The spectral information of ROS was recorded on an electron paramagnetic resonance spectrometer (EPR, JE-FA 300, Tokyo, Japan) using 50 mM DMPO as spin capture agents. The concentration of AMT in reaction samples was determined using reversed-phase high-performance liquid chromatography (HPLC) coupled with a variable wavelength ultraviolet detector (Jasco, Tokyo, Japan, λ = 205 nm) and a Spheri-5 C18 separation column. The isocratic mobile phase consisted of 65% (*v*/*v*) acetonitrile and 35% (*v*/*v*) water, delivered at a flow rate of 1.0 mL/min. Degradation products were analyzed using UPLC–MS/MS instrumentation (Xevo TQ-S, Waters, Milford, MA, USA), with an Agilent 6460 triple quadrupole system equipped with a Zorbax XDBC8 chromatographic column (2.1 mm × 150 mm × 3.5 μm) and operated in positive electrospray ionization mode (ESI+; m/z 50–600). The LC–MS mobile phase comprised 65% (*v*/*v*) acetonitrile and 35% (*v*/*v*) water, flowing at 1.0 mL/min.

## 4. Conclusions

This study developed an electrocatalytic persulfate (PS) activation system using carbon-coated ZnO (C@ZnO) electrodes, enabling 97.5% degradation of 5 mg/L amitriptyline (AMT) in 120 min under optimized conditions (200 C@ZnO anode, pH 7.0, 20 mA/cm^2^, 0.5 mM PS). Material characterization revealed that the amorphous carbon layer enhanced surface area (443.44 m^2^/g) and porosity, facilitating charge transfer and radical generation. EPR and quenching experiments showed that both acidic and neutral pH conditions were demonstrated to favorably enhance the electrocatalytic degradation efficiency by improving adsorption performance and inhibiting •OH decomposition, with ^•^OH and SO_4_^•−^ as predominant reactive species. UPLC–MS/MS analysis identified three AMT degradation pathways involving 13 intermediates, and sustained radical attacks achieved complete mineralization to CO_2_, H_2_O, and inorganic ions. The system efficiently degraded AMT under near-neutral pH (7.0) while avoiding toxic dealkylated byproducts. The electrode maintained >90% efficiency after five reuse cycles, providing a viable electrocatalytic strategy for antibiotic wastewater treatment and laying a foundation for advanced industrial purification technologies.

## Figures and Tables

**Table 1:** BET of Pristine ZnO, 50 C@ZnO, 100 C@ZnO, 200 C@ZnO, and 400 C@ZnO.

Sample	BET Surface Area (m^2^/g)	Pore Volume (cm^3^/g)	Pore Size (nm)
Pristine ZnO	4.04	0.012	12.054
50 C@ZnO	443.44	0.197	1.777
100 C@ZnO	319.45	0.150	1.879
200 C@ZnO	224.30	0.109	1.950
400 C@ZnO	169.20	0.085	2.008

## Data Availability

Not applicable.
